# “Local injection of freshly collected autologous adipose tissue as a novel adjunctive treatment in hidradenitis suppurativa”

**DOI:** 10.1016/j.jdcr.2026.06.007

**Published:** 2026-06-13

**Authors:** Mette M. Lange, Lilli Lundby, Helene T. Hougaard, Jørgen Agnholt, Anders Dige, Trine Bertelsen

**Affiliations:** a Department of Dermatology and Venereology, Aarhus University Hospital, Aarhus, Denmark; b Department of Surgery, Aarhus University Hospital, Aarhus, Denmark; c Department of Surgery, Regional Hospital Randers, Randers, Denmark; d Department of Hepatology and Gastroenterology, Aarhus University Hospital, Aarhus, Denmark; e Hudlægecenter Nord, Aalborg, Denmark

**Keywords:** autologous adipose tissue, hidradenitis suppurativa, mesenchymal stem cells, treatment

Hidradenitis suppurativa (HS) is a chronic inflammatory skin disease of the terminal follicular epithelium, with a worldwide prevalence of approximately 0.4%.^1^ It is characterized by inflammatory nodules and abscesses that may lead to the formation of persistent tunnels and scaring. Disease manifestations are typically symmetric with predilection sites, including the axillary, submammary, abdominal, inguinal, genital, and perianal regions. Pain is the predominant symptom; however, discharge, malodor, itch, and fatigue are also frequently reported by patients, with significant impairment on quality of life. Previous studies suggest that women are more often affected by HS than men, except in cases of perianal involvement, which demonstrates a male predominance.^2^^,^^3^

When HS is localized in the perianal region, Crohn disease (CD) should be considered as a differential diagnosis, particularly in the presence of fistulas or gastrointestinal symptoms. Previous studies have shown a significant association between HS and CD.^4^

Unlike other inflammatory skin diseases, surgical interventions play a key role in the treatment of HS, especially in moderate to severe disease where tunnels and scar tissue are present. Varies surgical techniques are used, including local and wide excision as well as deroofing, performed either with conventional surgery or CO_2_ laser.^3^^,^^5^

Local injections of adipose-derived stem cells (ADSCs) or freshly collected autologous adipose tissue (FCAAT) have demonstrated promising results in long-term healing of perianal fistulas in both patients with and without CD.^6^, ^7^, ^8^, ^9^ The underlying mechanism of action is not fully understood; however, it is thought to be a combination of local angiogenesis, anti-inflammation, immunomodulation, and tissue regeneration.^10^ These observations have led to the hypothesis that injection of FCAAT may also improve similar inflammatory changes seen in patients with HS. A systematic literature search using the PubMed database found no previous studies or case reports describing treatment with injection of ADSCs or FCAAT in patients with HS.

In this case series, we report 3 cases treated with local injection of FCAAT and evaluate its potential as a new adjunctive treatment in patients with HS.

## Cases

Between January 2020 and September 2024, 3 male patients, all with severe HS were treated with local injection of FCAAT.

Injections were administered in sites with chronic, irreversible changes in the axillary, inguinal, genital, or perianal regions. Each patient received between 2 and 3 treatments, with months to years apart (Table I).

**Table I:** Patient characteristics

Cases	Sex	Age at baseline, y	Debut	Family history of HS	Comorbidities	Hurley stage	Systemic treatment (previous and current)	Treatments with injection of autologous adipose tissue
Case 1	Male	53	Second decade	Yes (children)	Psoriasis, cicatricial alopecia, bipolar disease, previous overconsumption of alcohol	III	Doxycycline, clindamycin + rifampicin, adalimumab	2, with ≈ 56 months interval
Case 2	Male	42	Fifth decade	Yes (sister)	Adiposity, hypercholesterol, previous overconsumption of alcohol	III	Clindamycin + rifampicin, adalimumab, dicloxacillin	3, with ≈28 and 12 months interval, respectively
Case 3	Male	37	Second decade	Yes (uncle)	Acne, adiposity, ADD	III	Tetracycline, isotretinoin, clindamycin + rifampicin, prednisolone+ amoxicillin/ clavulanic acid + metronidazole + fluconazole, adalimumab, infliximab, bimekizumab	3, with ≈6 and 9 months interval, respectively

*ADD*, Attention deficit disorder; *HS*, hidradenitis suppurativa.

Harvesting and injection of autologous adipose tissue was performed in a single procedure in general anesthesia with the patient in lithotomy position. The first step comprised harvesting of subcutaneous adipose tissue from the lower abdomen. A mixture of Ringer solution and 1 mg of adrenaline was injected into the subcutaneous adipose tissue. Adipose tissue was collected via manual liposuction, then centrifuged, and the liquid fraction discarded.

The remaining adipose tissue was homogenized by shifting it between 2 syringes. The HS affected area was surgically revised, excising abscesses, nodules, and persistent tunnels. The microfragmented adipose tissue was then injected subcutaneously along the edges of the wound as the cannula was withdrawn. Wounds were left open for secondary healing. In one case, surgical revision or excision was not performed prior to injection.

Information on medical history, symptoms, complications, and treatment response was obtained retrospectively from medical records. Additionally, patient-reported outcomes were collected through a questionnaire using a 5-point Likert scale ranging from great improvement to great exacerbation to assess the effect of the procedure.

**Fig 1:**
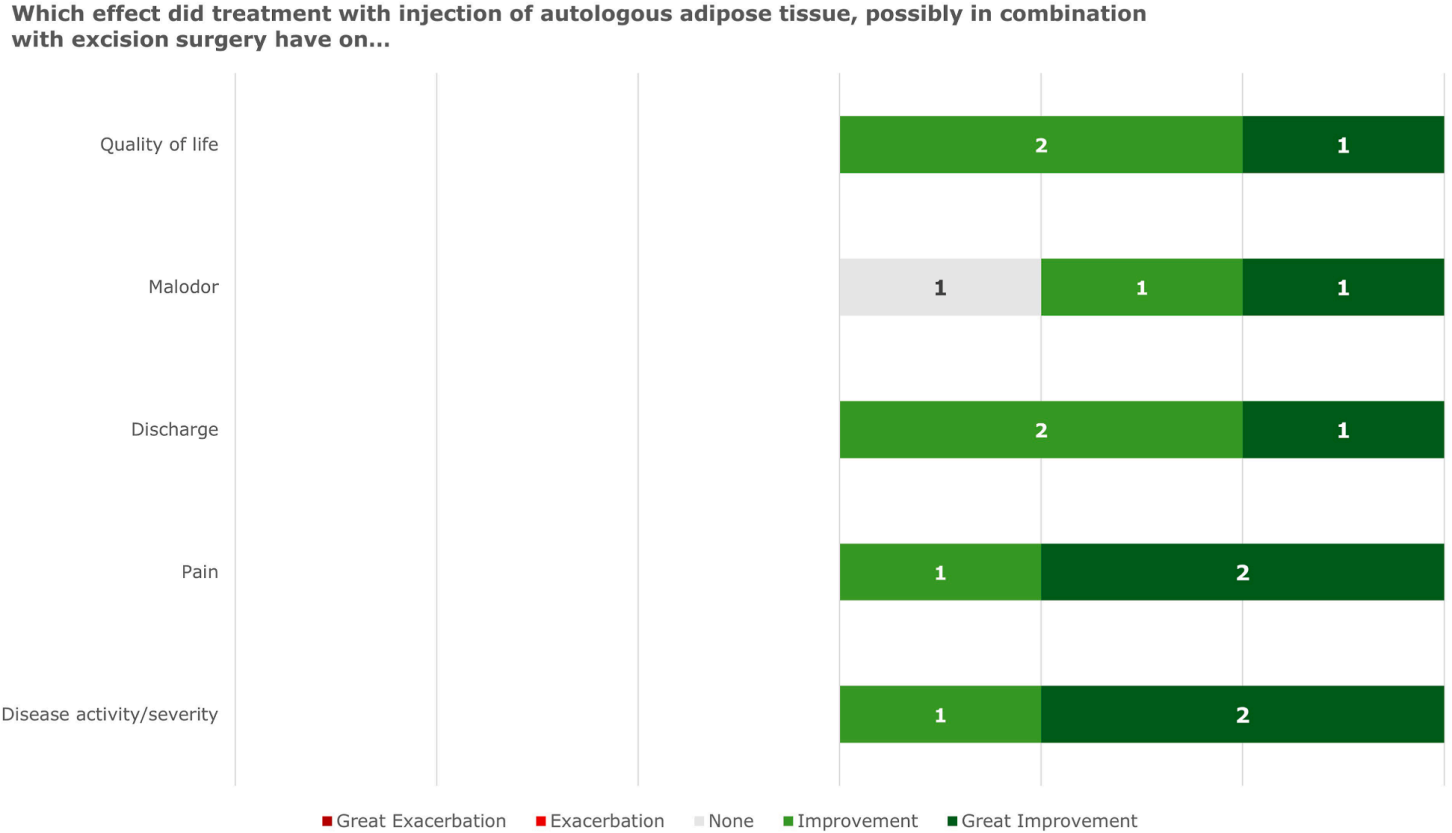
Questionnaire results.

All 3 patients reported either “improvement” or “great improvement” in disease activity/severity, pain, discharge, and quality of life. Two out of 3 patients also reported “improvement” or “great improvement” in malodor (Fig 1).

Complications were few and transient, including pain, hematoma, and hyposensitivity. All patients stated that they would undergo the procedure again if indicated.

**Fig 2:**
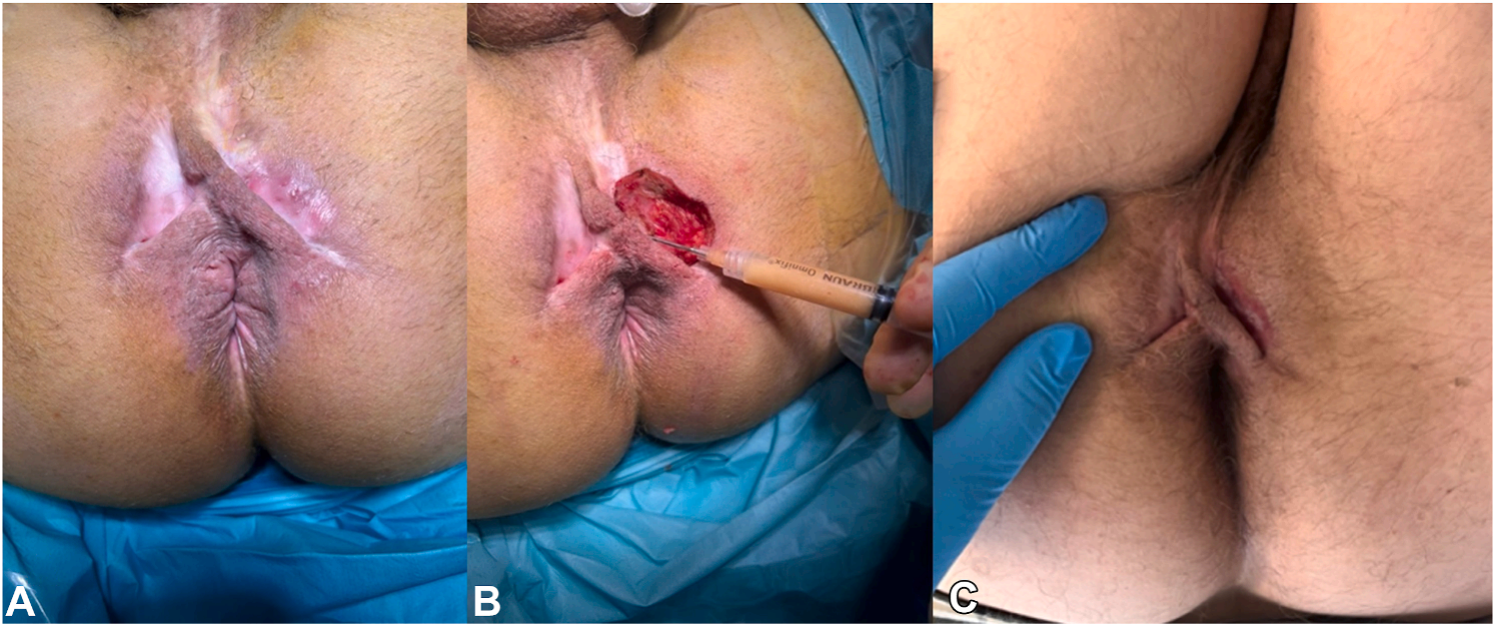
**A,** Before surgery. **B,** During surgery**. C,** Fifty-three days after surgery.

The patient-reported outcomes were supported by follow-up notes in the medical records reporting partial to great treatment response, with improvement in pain, discharge, hemorrhage, as well as softening of scar tissue (Fig 2). Time for follow-up showed great variation between 2 and 19 weeks.

## Discussion

The management of HS is complex and includes both topical-, intralesional-, systemic therapy and surgery, often in combination depending on clinical manifestation and severity. For several treatment modalities, evidence of efficacy is sparse because of a limited number of studies, with many of them being uncontrolled, retrospective, and with only a few patients included. This case series suggests a potential role for injection of FCAAT, as adjunctive treatment to conventional surgical and medical interventions, in the management of HS. All cases demonstrated a positive response to local injection of FCAAT, the 2 cases who underwent concomitant surgical revision/excision reported the best outcome. Injection of FCAAT is a simple, minimally invasive procedure and treatment was well tolerated, with only a few transient complications. The observed outcomes should be interpreted as preliminary, as the study has several limitations. The case series is retrospective with only 3 patients included, no control group or intrapatient control, and no randomization. Results of the study are based on patient-reported outcomes and clinician notes and lacks more standardized and objective outcome measures. Furthermore, the clinical outcomes observed can be affected by confounding variables such as natural fluctuations in disease activity and concurrent initiation or adjustment of medical therapies, including biologics. Despite its limitations, with this case series, we hope to encourage further investigation into the potential effect of local injection of FCAAT and/or ADSCs in patients with moderate to severe HS and the cellular and molecular mechanisms behind.

### Declaration of generative AI and AI-assisted technologies in the writing process

During the preparation of this work the authors used ChatGPT in order to check grammar, punctuation, and language to improve readability of the work. After using this tool, the authors reviewed and edited the content as needed and take full responsibility for the content of the publication.

## Conflicts of interest

None disclosed.
